# The Pathophysiology of Type 2 Diabetes Mellitus in Non-obese Individuals: An Overview of the Current Understanding

**DOI:** 10.7759/cureus.7614

**Published:** 2020-04-10

**Authors:** Idowu Olaogun, Mina Farag, Pousettef Hamid

**Affiliations:** 1 Endocrinology, University College Hospital, Ibadan, NGA; 2 Medicine, California Institute of Behavioral Neurosciences and Psychology, Fairfield, USA; 3 General Medicine, Solihull Hospital, Solihull, GBR; 4 Neurology, California Institute of Behavioral Neurosciences and Psychology, Fairfield, USA

**Keywords:** pathophysiology, non-obese, type2 diabetes mellitus

## Abstract

The pandemic of type 2 diabetes mellitus (T2DM) has been largely attributed to the increasing prevalence of worldwide obesity at a geometric rate. However, the number of non-obese patients with T2DM is also on the rise, and it is as high as 60-80% in some Asian countries. These non-obese individuals have certain peculiarities and have a higher mortality rate compared with obese individuals. The pathophysiology of T2DM in non-obese individuals remains poorly understood, and this has an impact on defining its management. This review discusses the current understanding of the pathophysiology of T2DM in non-obese individuals. The definition of T2DM in non-obese individuals remains controversial because of the limited clinical measurements, and the current definition of obesity using body mass index (BMI) is not very helpful as these individuals have BMIs of <25K g/m3, which is considered normal. Many authors have argued that the so-called non-obese people are actually metabolically obese; however, in terms of the measurements clinically available, they are non-obese. The simplistic understanding of the mechanism of the pathophysiology sees it in terms of the balance between insulin secretion and insulin resistance. The pathogenesis of insulin resistance in a lean patient has been proven to be the same as what is seen in an obese individual, but most studies confirm more severe functional insulin secretory defects in lean individuals compared to the obese phenotype. The mechanism underlying this form of T2DM is still poorly defined, and more research is required to understand the mechanism of sarcopenic obesity, which some studies have revealed.

## Introduction and background

Diabetes mellitus (DM) is a heterogeneous disease and hence lacks a precise definition. Its clinical features vary widely within and between populations [1]. It is a significant public health problem. The International Diabetes Federation (IDF) has projected that 629 million people will be diagnosed with type 2 DM (T2DM) globally by 2045, compared to 425 million in 2017 [2]. The exponential increase in the prevalence has been attributed predominantly to the obesity pandemic in over 80-90% of the cases [3,4]. However, it has been observed that 10-20% of individuals with T2DM are non-obese, after excluding latent autoimmune diabetes in adults (LADA) and other forms of diabetes. In some parts of the world, especially Asian countries, the prevalence of the non-obese variant is as high as 60-80% of the total T2DM burden [5]. Some studies also show that these individuals are at an increased risk of cardiovascular events than obese patients, and this makes it essential to arrive at a better understanding of the mechanisms of the pathophysiology of this condition [6].

The simplistic explanation for the pathophysiology of T2DM has linked it to the interplay between beta-cell dysfunction and insulin resistance, but the specific defects are complex and not fully understood [7]. Recently, DeFronzo et al. published a study that linked the pathophysiology of T2DM to its treatment. However, a lot still remains to be understood [8]. While insulin resistance superimposing on the defective insulin secretion has been described for T2DM in obese individuals, its pathophysiology in the non-obese has been a dilemma since most of the proposed models have been done in obese subjects. Some studies have shown that although these patients are not technically obese, they are metabolically obese and hence the same pathophysiology applies to them as well [9]. While this is true to some extent, there are a lot of differences in terms of the pathophysiology between the obese and non-obese patients that need to be clarified so that the management of these individuals can be better defined.

This review aims to discuss the current understanding of the pathophysiologic mechanism of non-obese T2DM with an emphasis on the lean subgroups.

## Review

Definition/measurement of obesity and its limitations

Obesity has overwhelmed both developed and developing countries (globesity) [10]. It is defined as abnormal or excessive fat accumulation in the body. It is crudely calculated in terms of BMI [3]. The need for precise measurement is important because of the deadly complications that the disease causes if left without intervention, especially with regards to cardiovascular diseases.

Three groups of individuals have been described as far as the T2DM phenotype is concerned. They are obese, patients with normal weight, and lean T2DM patients, with BMIs of >25, 18-25, and <18 kg/m2 respectively. The last two belong to the non-obese group, and the leanness of this group is not due to the disease itself or other pathological factors [11]. The different distinct T2DM phenotypes are characterized by a predominance of different metabolic defects and pathophysiologic mechanisms. The BMI measurement does not take sex differences in the distribution of fat and age-related decline in muscle mass into consideration. Thus, BMI results are an inaccurate assessment of the adiposity that the definition is meant to clarify [12]. A person with central obesity can have a normal BMI and yet can carry a high mortality risk. Although BMI correlates with the degree of insulin resistance linearly, it does not correspond to some degree of fatness in different populations due to different body proportions, i.e., the bodyweight vs. body shape in different ethnic groups.

Due to these limitations of BMI measurements, the crucial question is whether patients with normal weight are truly non-obese given that some of them (13% in one study by Mohan et al.) have abdominal obesity that is independent of the total obesity associated with insulin resistance, higher risk of microvascular complications, and increased cardiovascular risk, i.e., metabolically obese individuals as opposed to the BMI-measured obesity [13,14]. Currently, abdominal obesity could be measured using the waist circumference or waist-hip ratio (WHR). The available means of measurement fail to accommodate the last group who are neither obese based on BMI calculation nor have abdominal obesity. They are the lean T2DM patients whose pathophysiologic mechanism is still dilemmatic. Pathophysiologic heterogeneity is higher in this group, and this imparts particular challenges in terms of therapy. The low body weight does not reflect poor beta-cell function or loss of weight due to longstanding uncontrolled T2DM [15].

Even among these lean patients, abnormal fat pads in various parts of the body can be encountered, which still give them the tendency to be metabolically obese (ectopic fat deposition) [9]. The clinical presentation, as well as the profile of the complications, is different in these individuals compared to the obese and individuals with normal weight [16]. These patients also have a higher risk of mortality, the so-called obesity paradox, though this is still dilemmatic [17].

Considering the inadequacy of current measurements in distinguishing leanness, future studies should investigate the complex interaction between body composition, amount and distribution of adipose tissue, and physical functioning in determining the development of lean diabetes [18].

Etiopathogenesis

Lean diabetes could be a new pathogenic entity; but based on the available evidence, it is likely a variant of T2DM. A complete understanding of the causes of T2DM will require a better knowledge of the environmental and genetic molecular determinants of both insulin action and secretory function.

Environmental Factors

Some environmental factors have been linked to T2DM in the non-obese, most of which are supported by many observational studies. They are grouped into factors in-utero, early life exposure, and adult social risk factors including socioeconomic background, smoking, and alcohol consumption. Some animal studies show that a low-protein diet in early life leads to decreased beta-cell mass and insulinopenia. Multiple studies have shown an association between lean diabetes and malnutrition in the early years of life and poor socioeconomic status [19]. This type of diabetes is also more prevalent in the rural setting than the obese and non-obese diabetes [3].

Chronic alcohol consumption has also been linked to pancreatic beta-cell dysfunction and apoptosis [19]. Exposure to passive and active smoking has a positive and independent association with diabetes. In fact, the male preponderance of the disease could be due to the prevalence of these social factors as confirmed by many observational studies [18]. Emphasis on modifiable risk factors like smoking and alcohol abuse that may further accelerate beta-cell failure in lean individuals may prevent further progression of the disease [18].

These patients are neither clinically nor pathophysiologically related to LADA. The markers of autoimmune destruction of beta cells are comparable to what is seen in the general population [14].

Genetic Factors

The strongest clues for the T2DM in non-obese are the genetic factors, which vary from one region of the world to the other. Accurate dissection of the genetic underpinnings of the disease may inform specific pharmaceutical interventions that can correct the pathogenic imbalance driving the disease. Some studies confirm positive family history ranging between 16-25% in non-obese individuals with T2DM [20].

Previously identified genes are only indirectly related to T2DM risk through their influence on obesity and lifestyle [21]. A multinational study of patients with lean diabetes identified that 29 of 36 other known T2DM risk loci were more strongly associated with lean rather than with obese individuals [22,23]. Most of the identified genes were related to beta-cell functions. The genetic predisposition leads to a more fragile beta cell, which results in early destruction and apoptosis. Other studies have also demonstrated that the genetic markers of such fragility are more common in lean than in obese individuals with T2DM. Polymorphism of transcription factor FL2 gene and ATP-sensitive potassium channel Kir6.2 are also identified. Carriers of the latter are leaner and more insulin sensitive. One study also identified TCF7L2, HNF1B/HNF4A, KCNJ11, KNCJ15, also called Kir4.2, and C566T SNP [24]. TCF7L2 is also the most common gene for LADA. In a study in China, CDLAL1, CDKN2BAS, KCNQ1, TCF7L2, CDC123/CAMK1D, HHEX, and TCF2 were identified. Genetically, insulin resistance may also play a role in the pathogenesis, but this would rather be classified under other specific types of diabetes rather than T2DM in non-obese.

Pathophysiology

The exact cause of hyperglycemia in a patient with lean T2DM is yet to be fully elucidated. One strong idea that is becoming popular is the concept of sarcopenic obesity in which there is metabolic obesity from excess adiposity with decreased muscle mass. Failing beta cells cannot even cope with the small amount of insulin resistance that the lean body weight confers. Going along with this, it is reasonable to suggest that the percentage of fat to the total body weight is more important than the total body weight itself. The question is whether we should aim at the lower body weight or at the lower body adiposity to prevent diabetes; this is likely the critical point but to achieve this may be difficult [25]. Also, there is conflicting evidence of the benefits of weight loss in this group of patients. Practically speaking, this should be individualized. While some studies have shown that weight loss is significantly associated with a lower risk for diabetes both in obese and non-obese patients, others have reported adverse effects of losing weight in lean patients [26]. The major pathophysiology appears to be a rapid beta-cell failure due to higher prevalence and early initiation of insulin use. This has been supported by many studies [27,28].

Defective Insulin Secretion

As noted earlier, the cause is likely largely due to insulin secretion with little or no insulin resistance as suggested by many studies. The fasting C-peptides levels are found to be average between type 1 and type 2 diabetes; however, the circulating insulin levels at baseline and post-stimulation with insulin secretagogue are lower compared to obese patients. All these suggest a more severe beta-cell failure in the non-obese group compared to obese individuals. The beta-cell failure is likely functional rather than structural as it has been noticed that although beta-cell mass is reduced in both lean and obese patients, the reduction is with lower function in non-obese patients [29]. A study suggests that lean T2DM had a lower beta-cell function using homeostatic model assessment beta (HOMA-B), compared to obese patients, without any significant difference in insulin resistance [30].

Insulin secretion is tightly controlled by feedback from the beta cells, plasma glucose, incretin hormones, insulin sensitivity, and neuronal controls like hyperglycemia, obesity, hyperlipidemia, oxidative stress, inflammation, and amyloid deposition, which can accelerate the beta-cell loss. Tight control of accelerating factors may slow the progression [31]. The beta-cell abnormalities develop over a long period, involving various steps including genetic predisposition, early and progressive beta-cell dysfunction, and loss and developments of metabolic abnormalities, which trigger apoptosis and sometimes autophagy in the absence of any regenerative mechanism of the beta cells. Further studies are still needed to establish this fact and the factors that lead to the destruction of beta cells.

The earliest and most consistent defect of insulin secretion is a loss of first-phase insulin secretion in response to intravenous glucose. The decreased insulin secretion is partly due to decreased beta-cell function mass seen in both lean and obese individuals and additional functional defects of pancreatic insulin secretion, leading to blindness and hyperglycemia, which is more marked in the non-obese group. Another relatively known defect contributing to the impaired insulin secretion in T2DM is the reduced secretion of the gut incretin hormones. The BMI and the WHR are oppositely directed to increase glucagon-like peptide-1 (GLP-1). It has been found that only non-obese patients with T2DM as defined by BMI have preserved GLP-1 secretion in response to an oral glucose load [32].

Insulin Resistance

Insulin resistance has been demonstrated in lean individuals, but mechanisms contributing to it are not well established. Hollenbeck et al. have demonstrated that although lean individuals could have both hepatic and peripheral resistance to insulin, it is not to the same extent as in obese patients with T2DM [33]. This has been directly estimated by the homeostatic model assessment for insulin resistance (HOMA-IR), and indirectly by triglyceride/high-density lipoprotein ratio (TG/HDL). Patients with insulin resistance have significantly higher TG levels, higher low-density lipoprotein (LDL) cholesterol, and lower HDL cholesterol levels compared with those with normal insulin sensitivity. The molecular cause of insulin resistance in the non-obese is still linked to lipids accumulation just as in obese patients. Masharani et al. have reported that insulin resistance in non-obese subjects is associated with activation of the c-Jun N-terminal kinase (JNK) pathway and impaired insulin signaling in skeletal muscle. Implicated in this disruption of cellular insulin action are the accumulation of lipids within skeletal muscles and the greater degree of overall adiposity that was observed in the insulin-resistant subjects [34]. The particular fat content of the body that leads to this has been demonstrated to vary based on racial origins as there is no conclusive study to affirm if it is the subcutaneous fat or visceral fat. While some studies have implicated either of the two, some recent studies have demonstrated both to be equally important, and further studies are still needed to establish this. Banerji et al. have demonstrated that visceral but not subcutaneous abdominal fat volume is associated with insulin resistance in black populations with T2DM [35]. Abate et al. have demonstrated that subcutaneous but not the visceral intraperitoneal or retroperitoneal fat volume is associated with insulin resistance in non-Hispanic whites with T2DM [36]. A study by Taniguchi et al. has demonstrated that insulin resistance is independently associated with subcutaneous and visceral fat areas in non-obese Japanese T2DM patients [37].

## Conclusions

The mechanism that underlies T2DM in non-obese individuals is yet to be elucidated. The current evidence for insulin-secretion defects is stronger than insulin-action defects. More research is needed to understand the mechanism of sarcopenic obesity and its implications for the treatment of this particular subset of T2DM.
